# A Rare Case of Rapidly Transforming Pancreatitis With Life-Threatening Complications and Multi-Organ Failure

**DOI:** 10.7759/cureus.16766

**Published:** 2021-07-30

**Authors:** Ibrahim A Ahmed, Candace Grifith, Sean Lipshutz, David Weinstein, Ravindra Hallur

**Affiliations:** 1 Internal Medicine, Mercy Catholic Medical Center, Philadelphia, USA; 2 Internal Medical, Philadelphia College of Osteopathic Medicine, Philadelphia, USA; 3 Internal Medicine, Drexel University College of Medicine, Philadelphia, USA; 4 Internal Medicine, University of Pennsylvania Perelman School of Medicine, Philadelphia, USA

**Keywords:** acute necrotizing pancreatitis, acute respiratory distress syndrome [ards], continuous renal replacement therapy (crrt), splenic vein thrombosis, acute pancreatitis, fungemia, hemorrhagic pancreatitis

## Abstract

Acute pancreatitis affects approximately 50,000-80,000 Americans each year. Eighty percent of those cases are related to alcohol use or biliary stones. It has traditionally been thought of as a self-limiting disease, in which the pancreas fully recovers with little or no intervention. The occurrence of complications, particularly infectious ones, characterize the most severe forms of acute pancreatitis and is associated with high mortality. We present a case of acute pancreatitis with transformation into necrotizing and hemorrhagic pancreatitis complicated with splenic vein thrombosis (SVT), acute respiratory distress syndrome (ARDS), acute kidney injury (AKI), and later, fungemia.

## Introduction

Acute pancreatitis is a condition in which the pancreas becomes inflamed over a short period of time. Some causes of pancreatitis include gallstones, ethanol, trauma, steroids, infection with mumps, malignancy, autoimmune etiology, scorpion sting, or idiopathic causes. Most people with acute pancreatitis present with upper abdominal pain worsening postprandially with radiation to the back, nausea, vomiting, and tachycardia. Mild cases may self-resolve. In more serious cases, hospitalization and treatment with bowel rest, intravenous fluids, anti-emetics, and analgesia are warranted. We present a case of alcohol-induced acute pancreatitis with transformation to both necrotizing and hemorrhagic pancreatitis and a multitude of major complications including splenic vein thrombosis (SVT), acute respiratory distress syndrome (ARDS), acute kidney injury (AKI), and fungemia.

## Case presentation

We present a 41-year-old male with a past medical history of hypertension and type 2 diabetes mellitus who presented to the emergency department with complaints of acute onset severe abdominal pain. He reported sharp, non-radiating epigastric abdominal pain, 10/10 in severity, that had been constant since the morning of admission. The epigastric pain was associated with nausea and one episode of non-bloody vomiting. The patient endorsed a history of chronic alcohol abuse for the past 20 years, with his last drink being the day prior to presentation. He drank approximately one bottle of tequila and 24 beers over the course of one day. He denied fevers, chills, chest pain, shortness of breath, diarrhea, and urinary symptoms.

**Figure 1:**
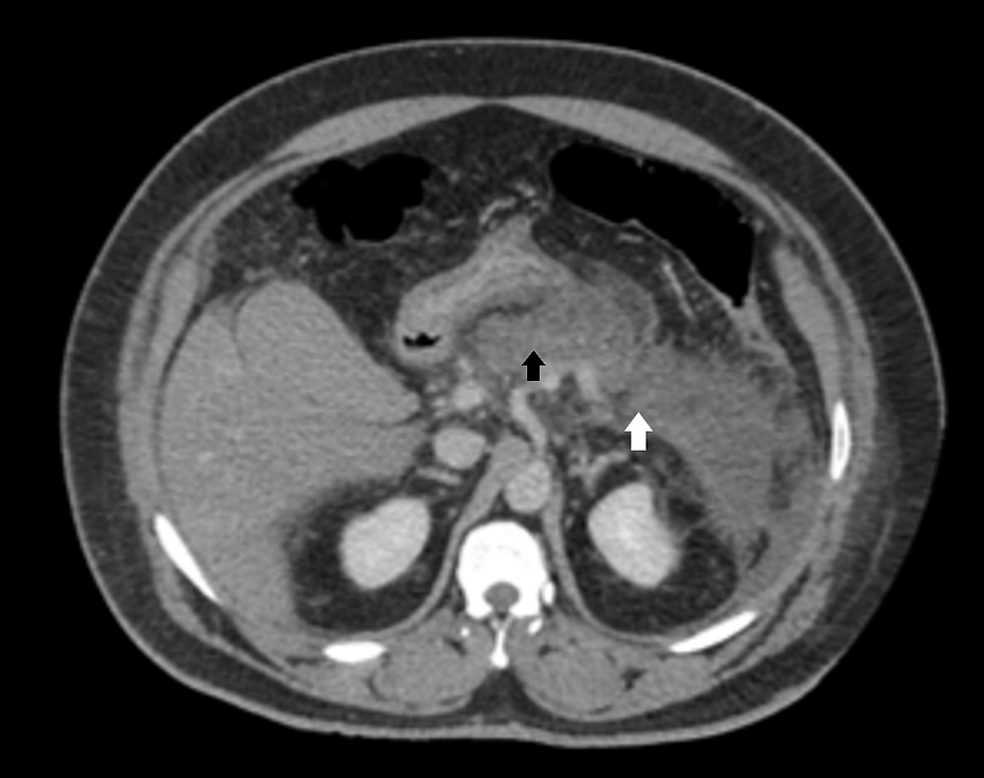
Severe acute interstitial edematous pancreatitis without organized peripancreatic fluid collection. Black arrow: pancreatitis; white arrow: peripancreatic fluid collection.

In the emergency department, vitals revealed a BP of 156/96 mmHg, pulse 104 bpm, respiratory rate 20 breaths/min, temperature 98.2 °F, with an O_2_ saturation of 100% on room air. Labs were significant for a lipase of 1948 U/L, white blood cell count of 20.7 thou/µL, magnesium of 1.6 mg/dL, bicarbonate of 14 mmol/L, and an anion gap of 27. The urine drug screen was positive for cocaine and opiates. A preliminary read of CT abdomen and pelvis revealed severe acute pancreatitis (Figure 1).

Lactate was markedly elevated to 10 and he was admitted to the ICU for the management of acute severe pancreatitis, lactic and metabolic acidosis, alcohol withdrawal, and electrolyte abnormality. He was started on Metronidazole and Cefepime and received aggressive IV fluid hydration.

**Figure 2:**
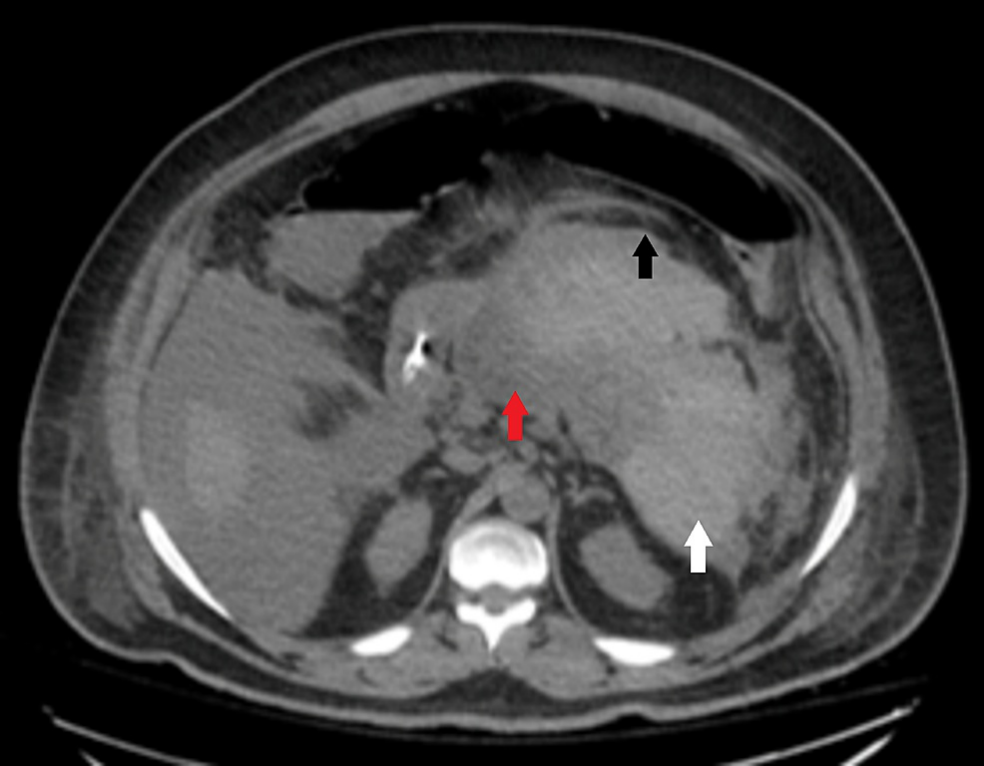
Hemorrhagic changes within the distal pancreatic body and tail with mass effect on the gastric body. Areas of necrosis suboptimally assessed in the absence of intravenous contrast. Red arrow: acute pancreatitis; white arrow: hemorrhagic changes within distal pancreatic body and tail; black arrow: mass effect on the gastric body.

Shortly after arrival to the tertiary center, the patient developed shock and obtundation due to the transformation of his necrotizing pancreatitis to hemorrhagic pancreatitis (Figure 2). He was transferred to the ICU, intubated, required four pressors, and placed on a massive transfusion protocol where he received 7 units packed red blood cells, 6 units of fresh frozen plasma, and one unit of platelets. He was subsequently taken to interventional radiology for embolization of a pancreatic branch artery that was actively extravasating. He was placed on continuous renal replacement therapy (CRRT) for renal failure and acidosis in the setting of shock. He developed severe ARDS and was paralyzed and proned for two sessions. An insulin drip was required after hyperglycemia developed in the setting of stress dose steroids.

**Figure 3:**
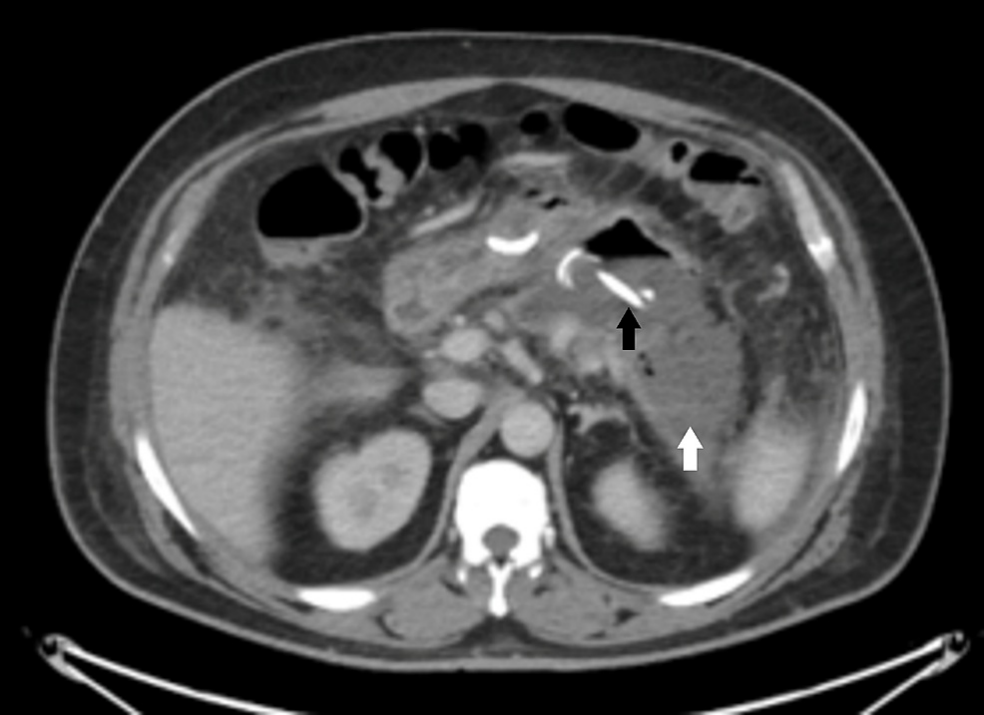
Interval placement of cyst gastrostomy with traversing pigtail drainage catheter with a decrease in the peripancreatic collection and expected air. Black arrow: traversing pigtail drainage; white arrow: decrease in the peripancreatic collection.

Ventilatory support was weaned and the patient was successfully extubated eight days later and taken off of CRRT. He was initially placed on Meropenem, later transitioned to Zosyn but was still noted to spike fevers. Infectious workup, due to the presence of diarrhea, was negative for *Clostridium difficile*, Campylobacter, and Shigella. Pancrelipase was initiated for ongoing diarrhea. After 11 days, he was stable for a downgrade to the general medical floors. At that time, blood cultures returned growing Gram-positive Cocci in clusters, later speciated to *Staphylococcus epidermidis*, and antibiotic treatment was adjusted and he was placed on Vancomycin for methicillin-resistant *Staphylococcus aureus* (MRSA) coverage. Repeat cultures started growing *Candida glabrata* and Caspofungin was added to his antibiotic regimen. Transthoracic echocardiography (TTE) and transesophageal echocardiography (TEE) were both negative for valvular vegetations. After discussions between interventional GI, ID, and IR, it was decided to pursue a cyst gastrostomy to identify and isolate the source of his fungemia (Figure 3).

## Discussion

**Figure 4:**
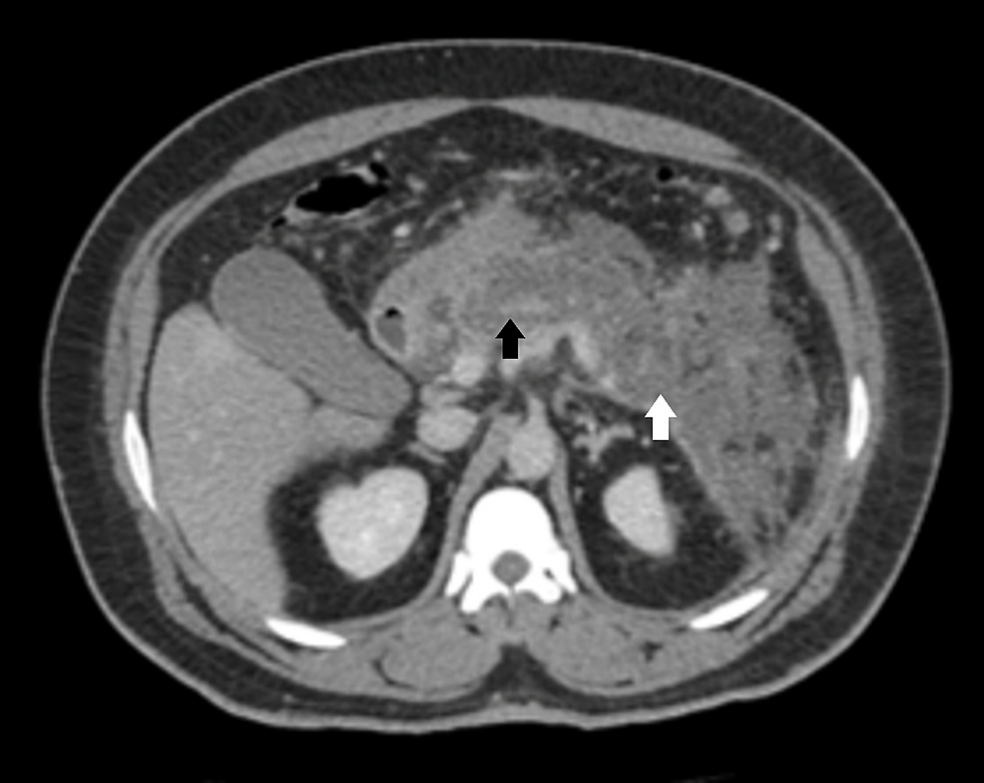
Acute necrotic pancreatitis with approximately 90% pancreatic parenchymal necrosis. Acute peripancreatic necrotic fluid collections measuring up to 7.9 cm with marked inflammatory changes surrounding the pancreas. Black arrow: 90% necrosis of the pancreas; white arrow: acute peripancreatic necrotic fluid collection.

Acute necrotizing pancreatitis accounts for 5-10% of acute pancreatitis cases. It is diagnosed when more than 30% of the pancreas is affected by necrosis [1]. This patient had 90% necrosis of the pancreas (Figure 4). Prognosis depends on whether the necrosis is sterile or infected with mortality rates of 5-10% and 20-30%, respectively. One rare complication of necrotizing pancreatitis is hemorrhage and hemorrhagic shock. Hemorrhagic disease is estimated to occur in 1% to 6.2% of patients with acute pancreatitis. The bleeding may occur in the gastrointestinal tract, peritoneal cavity, or from fluid collections in the pancreatic parenchyma [2]. Once hemorrhagic transformation occurs, there are limited treatment options. Angiography with embolization is considered first-line while surgery is used in refractory cases [2]. In our case, the patient’s hemodynamics rapidly deteriorated once the pancreatic necrosis was detected, his hemoglobin decreased from 8.5 to 6.7 g/dL, and the patient required admission to medical intensive care unit (MICU) for hemodynamic support, requiring four vasopressors and a massive transfusion protocol. Angiography by interventional radiology demonstrated active extravasation from multiple branches of the pancreatic Magna artery. Bleeding was controlled with coil embolization of the pancreatic Magna artery, and surgery was therefore not required. Hemoglobin remained stable at 13 and lactate normalized from 19.2 post-embolization, however, the patient continued to require vasopressor support. Prognosis is very poor with hemorrhagic transformation with mortality rates estimated as high as 40-50% [3].

Splenic vein thrombosis

Pancreatitis-induced SVT is a well-noted complication in the literature with an incidence rate noted to be 22.6% in acute pancreatitis in a recent meta-analysis [4]. The pathogenesis is multifactorial including local inflammation, venous stasis from enlarged pancreatic parenchyma/pseudocyst, and systemic activation of the coagulation cascade [5]. Although there are no official guidelines for use of anticoagulation for SVT in the setting of acute pancreatitis, management is usually based on resolving underlying acute pancreatitis and anticoagulation [6]. While it is true that anticoagulation has been shown to increase rates of thrombus resolution, clinical judgment of risk-benefit must be made as anticoagulation may compound the effects of coagulopathy from shock liver and exacerbate GI bleeding [7]. This patient was initially started on heparin drip but it was stopped and reversed with protamine once hemorrhagic shock was evident. Anticoagulation was subsequently held for the remainder of the admission. The SVT had resolved by approximately one month. Of note patient did develop a new pulmonary embolism and DVT in bilateral peroneal veins towards the end of his admission. An IVC filter was placed but anticoagulation was not started due to the recent history of GI bleed.

Acute kidney injury

The patient developed oliguric AKI. In the 72 hours, he developed hemorrhagic shock, his urine output was 745 mL (approximately 10 liters input), serum creatinine rapidly rose from 1.8 to 5 and the patient became markedly volume overloaded. The etiology of his AKI was likely multifactorial, and the differential diagnosis includes AKI secondary to ischemic acute tubular necrosis in the setting of hemorrhagic shock versus secondary to nephrotoxin exposure (IV contrast for CT scans). It was likely further exacerbated by the use of vasopressors [8]. The patient was initially started on continuous venovenous hemodialysis (CVVHD), a type of CRRT, through a non-tunneled dialysis catheter, however, the circuit clotted hours into the dialysis session. The patient was subsequently started on continuous venovenous hemofiltration (CVVH) which was complicated by circuit clotting after several hours. Normally in CRRT, systemic anticoagulation with unfractionated heparin is used to prolong the life of the extracorporeal circuit [9]. However, anticoagulation was contraindicated in this patient in the setting of active GI bleeding. The decision to use CRRT as opposed to intermittent hemodialysis in this patient was based on his marked hemodynamic instability and volume overload. Hypotension is less common with CRRT as fluid and solutes are removed at a slower pace over a longer period compared to intermittent hemodialysis [10]. However, several randomized controlled trials have shown no difference in mortality or recovery of kidney function between CRRT and intermittent hemodialysis [11]. Furthermore, early CRRT in the setting of acute pancreatitis has been suggested to increase the elimination of pro-inflammatory cytokines and autodigestive enzymes. Although this was not our indication for starting CRRT in this patient, we believe that it played an anti-inflammatory role in our patient [12]. We also administered furosemide 80 mg IV once with the initiation of CRRT to augment urine output and started 40 mg IV daily thereafter. Over the next few days, kidney function dramatically improved, and urine output normalized. The patient did not require further RRT.

Acute respiratory distress syndrome

ARDS in the setting of acute pancreatitis occurs in 0.2% cases of acute pancreatitis and carries a mortality rate of 11% [13]. Diagnostic criteria of ARDS include bilateral pulmonary infiltrates on radiologic imaging not fully explained by heart failure or volume overload and PaO_2_/FiO_2_ < 300 mmHg, with severe ARDS, indicated with p/f ratio <100 mmHg [14]. This patient developed ARDS one day after being intubated and started on mechanical ventilation. His p/f ratio was <300 and chest X-ray showed bilateral infiltrates consistent with ARDS. Management approach to ARDS usually includes a combination of maintaining high positive end-expiratory pressure (PEEP), muscle relaxants, recruitment maneuvers, pulmonary protective strategies, and venovenous extracorporeal membrane oxygenation [15]. In our patient, we reduced tidal volume from 7 cc/kg to 6 cc/kg, maintained plateau pressures <30 cmH_2_O. Despite these efforts, the patient’s hypoxemia worsened and we paralyzed the patient with cisatracurium and proned (in total 2 × 13 hour sessions). It should be noted that the pulmonary infiltrates could in part be explained by pulmonary edema as the patient was markedly volume overloaded, and the pulmonary edema improved after CRRT and administration of furosemide. The patient’s respiratory status subsequently improved, with an improving p/f ratio to >200 and pulmonary infiltrates. The patient was extubated seven days later and transitioned to a high flow nasal cannula and was saturating well on 40L FiO_2_ 40%.

## Conclusions

In this case report, we present an unusual case of severe acute necrotizing pancreatitis with numerous critical complications such as hemorrhagic shock, splenic venous thrombosis, ARDS, and sepsis. We highlight the rarity and the rapid rate with which these complications develop and our multidisciplinary approach in managing these complications as they occur. It also highlights the importance of clinical judgment and risk-benefit assessment in managing these complications as each case is unique.
